# Pan-cancer analysis of the prognostic and immunological roles of *DEAD-box helicase 5* (*DDX5*) in human tumors

**DOI:** 10.3389/fgene.2022.1039440

**Published:** 2022-10-13

**Authors:** Shixuan Liu, Yanbin Liu, Xi Zhang, Xuanlin Song, Boxiang Zhang, Yong Zhang

**Affiliations:** ^1^ Department of Thoracic Surgery, The First Affiliated Hospital of Xi’an Jiaotong University, Xi’an, China; ^2^ Health Science Center, Xi’an Jiaotong University, Xi’an, China

**Keywords:** DDX5, pan-cancer, biomarker, prognosis, immunothearpy, methylation, phosphorylation

## Abstract

**Background:** Recent studies have demonstrated the significance of the *DEAD-box helicase 5* (*DDX5*) gene, which is involved in pathways concerning the modification of RNA structures. *DDX5* functions as a coregulator of cellular transcription and splicing, and participates in the processing of small noncoding RNAs. The aberrant regulation of *DDX5* expression possibly plays a significant role in the genesis of cancer. However, there are no comprehensive pan-cancer studies on *DDX5*. This study is the first to conduct a pan-cancer analysis of *DDX5* for aiding the diagnosis and treatment of cancer.

**Methods:** The gene expression, genetic alterations, protein phosphorylation, promoter methylation, immune infiltration, and enrichment analyses of *DDX5* were performed using data retrieved from The Cancer Genome Atlas (TCGA), Genotype-tissue Expression (GTEx), Human Protein Atlas (HPA), Tumor Immunological Estimation Resource 2.0 (TIMER2.0), Gene Expression Profiling Interactive Analysis (GEPIA), DNA methylation interactive visualization database (DNMIVD), and Search Tool for the Retrieval of Interaction Genes/Proteins (STRING). Data analyses were performed with the R software and other webtools.

**Results:** The expression of DDX5 mRNA decreased significantly in 17 cancer types, but increased significantly in eight cancer types. The enhanced expression of DDX5 mRNA in the tumor samples was related to decreased overall survival (OS), progression-free interval (PFI), and disease-specific survival (DSS) in three cancers, but increased OS, PFI, and DSS in other cancers. The DNA promoter methylation level was significantly reduced in eight cancer types, and there were exceptions in the methylation levels of the *DDX5* promoter in four cancer types. The expression of DDX5 mRNA was highly correlated with the infiltration of CD8^+^ T cells, cancer-associated fibroblasts, and B cells in a wide variety of malignancies. The findings revealed a strong association between *DDX5* and its co-expressed genes in numerous cancer types. Enrichment analysis suggested that *DDX5* was associated with multiple cellular pathways, including RNA splicing, Notch signaling pathway, and viral carcinogenesis, which was consistent with the results of previous studies.

**Conclusion:** The findings obtained herein provide further information on the oncogenic potential of *DDX5* in diverse tumor types. We propose that *DDX5* has important roles in tumor immunity and the diagnosis of cancer.

## Introduction

Cancer is the leading cause of death worldwide and significantly affects the quality of life of patients, and imposes a considerable burden on global health and economy (Sung et al., 2021; Siegel et al., 2022). At present, there are no therapeutic regimens for the complete eradication of cancer. In recent years, bioinformatic tools have become essential for the analysis of genes associated with the incidence of cancer (Kasaian et al., 2014). Owing to the complexity of the mechanisms underlying the occurrence of tumors, pan-cancer analysis is crucial for determining the principle of carcinogenesis. The current publicly available databases are highly advanced, and bioinformatics resources, including the Gene Expression Omnibus (GEO) database and The Cancer Genome Atlas (TCGA), provide adequate data for functional genomic studies on all types of cancers (Barrett et al., 2013; Tomczak et al., 2015; Blum et al., 2018). In this study, the association between the expression of *DEAD-box helicase 5 (DDX5)* and the incidence of various cancers was determined using the R software and other web datasets.

The DDX5 protein is often referred to as p68 and is a member of the DEAD box family of RNA helicases. The p68 protein is a potent oncogenic biomarker and a therapeutic target for cancer (Li et al., 2021). Previous studies have established the overall biochemical features of DDX5 and its extensive role in cellular metabolism, including alternative pre-mRNA splicing (Lee et al., 2018), DNA replication (Mazurek et al., 2012), DNA damage (Nicol et al., 2013), ribosome biogenesis (Jalal et al., 2007; Saporita et al., 2011), miRNA biogenesis (Davis et al., 2008; Wang et al., 2012; Hong et al., 2013; Dardenne et al., 2014), and transcriptional regulation (Dardenne et al., 2014). In addition, studies have indicated that *DDX5* plays a crucial role in tumorigenesis in a wide range of malignancies (Janknecht, 2010), including hepatocellular carcinoma (Xue et al., 2018; Mani et al., 2020), breast cancer (Hashemi et al., 2019), prostate cancer (Taniguchi et al., 2016), and thyroid cancer (Lan et al., 2022), and functions in numerous signaling pathways, including the Wnt/*β*-Catenin signaling pathway (Wang et al., 2015) and the Akt signaling pathway (Xue et al., 2018). An increasing number of studies are conducted on a yearly basis on *DDX5*; however, thorough analysis of the functions of *DDX5* in carcinogenesis using bioinformatics tools has not been performed to date. This study is the first to perform pan-cancer analysis of the *DDX5* gene using data from TCGA, webtools, and the R software.

In this study, analyses of *DDX5* expression, Kaplan-Meier survival, clinical relevance, DNA promoter methylation, DNA alteration, phosphorylation, immune infiltration, and enrichment analyses were performed. The results of these analyses revealed several differences between tumor and normal matched tissues in terms of the aforementioned parameters of the *DDX5* gene that could play a crucial role in tumorigenesis. The probable mechanisms underlying the roles of *DDX5* in the occurrence and progression of cancer and clinical prognosis were additionally explored.

## Materials and methods

### Analyses of *DEAD-box helicase 5* mRNA and protein expression

Data pertaining to the differential expression of DDX5 mRNA in different cancer types and normal matched tissues were retrieved from TCGA and GTEx using R version 3.6.3, and visualized using the ggplot2 package in R. The Ualcan online tool (http://ualcan.path.uab.edu/index.html) was used for analyzing protein phosphorylation and expression (Chandrashekar et al., 2017). DNA methylation was analyzed using DNMVID (http://119.3.41.228/dnmivd/) (Ding et al., 2020). It is crucial to determine the expression of DDX5 protein in several organs. We therefore used the Human Protein Atlas (HPA) database (https://www.proteinatlas.org/) for analyzing the expression of DDX5 protein in different organs. The expression data were transformed using the log_2_(TPM + 1) normalization method for subsequent analysis. The Wilcoxon rank sum test was used for determining the significance; *p* < 0.05 was considered to be statistically significant. Outlier analysis was performed during statistical analysis. The clinical relevance between the expression of *DDX5* and the T-stage of tumors was also estimated based on the data obtained from TCGA using R, version 3.6.3.

### Association between survival prognosis and *DEAD-box helicase 5* expression

Survival prognosis was analyzed using Kaplan-Meier curves for determining the association between the mRNA expression of DDX5 and the prognosis of cancer, measured in terms of overall survival (OS), progression-free interval (PFI), and disease-specific survival (DSS), for 33 types of tumors in TCGA. A cutoff of 50% expression was used for separating the data into high and low expression categories. The expression data were transformed using the log_2_(FPKM + 1) normalization method for subsequent analysis. We used the log-rank test for analyzing the level of significance. Univariate survival analysis was performed for determining the 95% confidence intervals and HRs (Hazard Ratios).

### Association between immune infiltration and *DEAD-box helicase 5* expression

We initially determined the relationship between immune infiltration and the expression of DDX5 mRNA across 24 immunocytes in 32 types of tumors and the findings were displayed as a heatmap. We additionally determined the correlation between the immune infiltrates and the expression of DDX5 mRNA using TCGA data, with the online TIMER 2.0 tool (http://timer.cistrome.org/) (Li et al., 2020). The relevant immune infiltrate cells were selected using the immunological association module of TIMER 2.0 and the search term “DDX5” in the “Gene Expression” function. Immune infiltration was estimated using the TIMER, CIBERSORT, CIBERSORT-ABS, QUANTISEQ, XCELL, MCPCOUNTER, and EPIC algorithms. The obtained information was illustrated with three heatmaps and scatter plots. A purity-adjusted version of Spearman’s rank correlation was used for calculating the *p*-values and partial correlation coefficients.

### Analyses of the genetic alterations of *DEAD-box helicase 5*

The genetic alterations of *DDX5* were evaluated using the cBioPortal webtool (http://cBioPortal.org) (Gao et al., 2013). The search term “DDX5” was entered in the “Quick Search” function for obtaining an overview of the genetic modifications of *DDX5*. The structural variants, alteration frequency, copy number alterations (CNAs), and mutation data of *DDX5* in 32 tumor types were obtained from the summary. Information regarding the mutations in *DDX5*, including the types and frequency of mutations, were determined from the “Mutations” module, and the tertiary structure of the DDX5 protein was schematically depicted. The most frequent site of mutation was selected, and its position was identified in the schematic diagram. The OS, DFI (disease-free interval), and PFI were estimated from the “Comparison/Survival” module for all cancer types in TCGA with varying degrees of genetic mutations in *DDX5*. The relevant data were graphically presented using the Kaplan-Meier plotter, and the log-rank test was used for determining the statistical significance.

### Enrichment analysis of *DEAD-box helicase 5*

The STRING webtool (https://string-db.org/) (Szklarczyk et al., 2021) was used for constructing a DDX5-based network of protein-protein interactions. The DDX5 gene network was analyzed using the DDX5 protein as the search term, and the search results were narrowed down to *Homo sapiens* for extracting the data for the DDX5 gene in humans. The number of interactors was first restricted to <10 and the minimum necessary score of interactions was set to low confidence for determining the principal interactions. The experimentally relevant DDX5-binding proteins were finally identified by capping the number of visible interactors at 50 and restricting the resources to “experiments” for expanding the network. The GEPIA 2.0 tool was used for identifying the target genes that were related to DDX5. The “Similar Gene Detection” module of the expression analysis tool was selected for analysis, and the search term “DDX5” was used to search for the 100 most similar genes in all the tumor tissues in TCGA (Cui et al., 2020). Additionally, the “correlation analysis” module of the GEPIA 2.0 webtool (http://gepia2.cancer-pku.cn) (Tang et al., 2017) was used for performing a Pearson correlation analysis between DDX5 and genes related to DDX5, and the results were illustrated with a dot plot following normalization with the log2TPM method. The association between DDX5 and other genes in the different cancer tissues in TCGA was determined using the TIMER2.0 webtool. The results were depicted with a heatmap comprising the purity-adjusted partial Spearman’s rho value as the correlation *p*-value in the purity-adjusted Spearman’s rank correlation test.

In order to compare the genes encoding proteins that bind to DDX5 with the genes that interact with DDX5, we identified the genes at the intersection of these two sets with a Venn diagram. KEGG pathway and GO enrichment analyses were subsequently performed for the genes encoding proteins that bind DDX5 and the genes that interact with DDX5. Statistical analyses were performed in two steps, including the conversion of Gene IDs followed by enrichment analysis, in which p.adj < 0.05 and q value < 0.2 were considered to be statistically significant. The results were visualized using the ggplot2 package in R, version 3.6.3.

## Results

### Analysis of *DEAD-box helicase 5* expression

**FIGURE 1:**
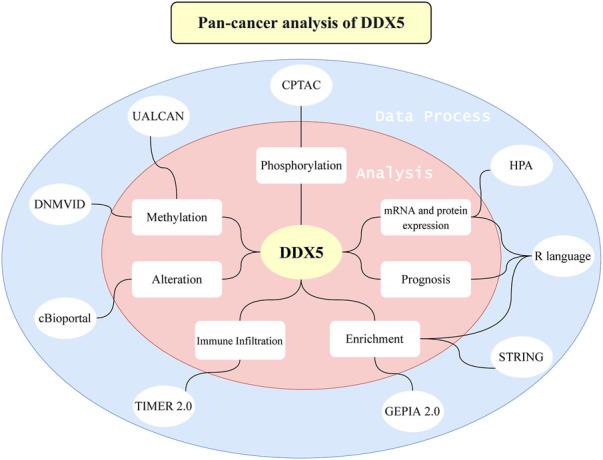
An overview about the whole study. A series of methods and webtools are applied to the study.

**FIGURE 2:**
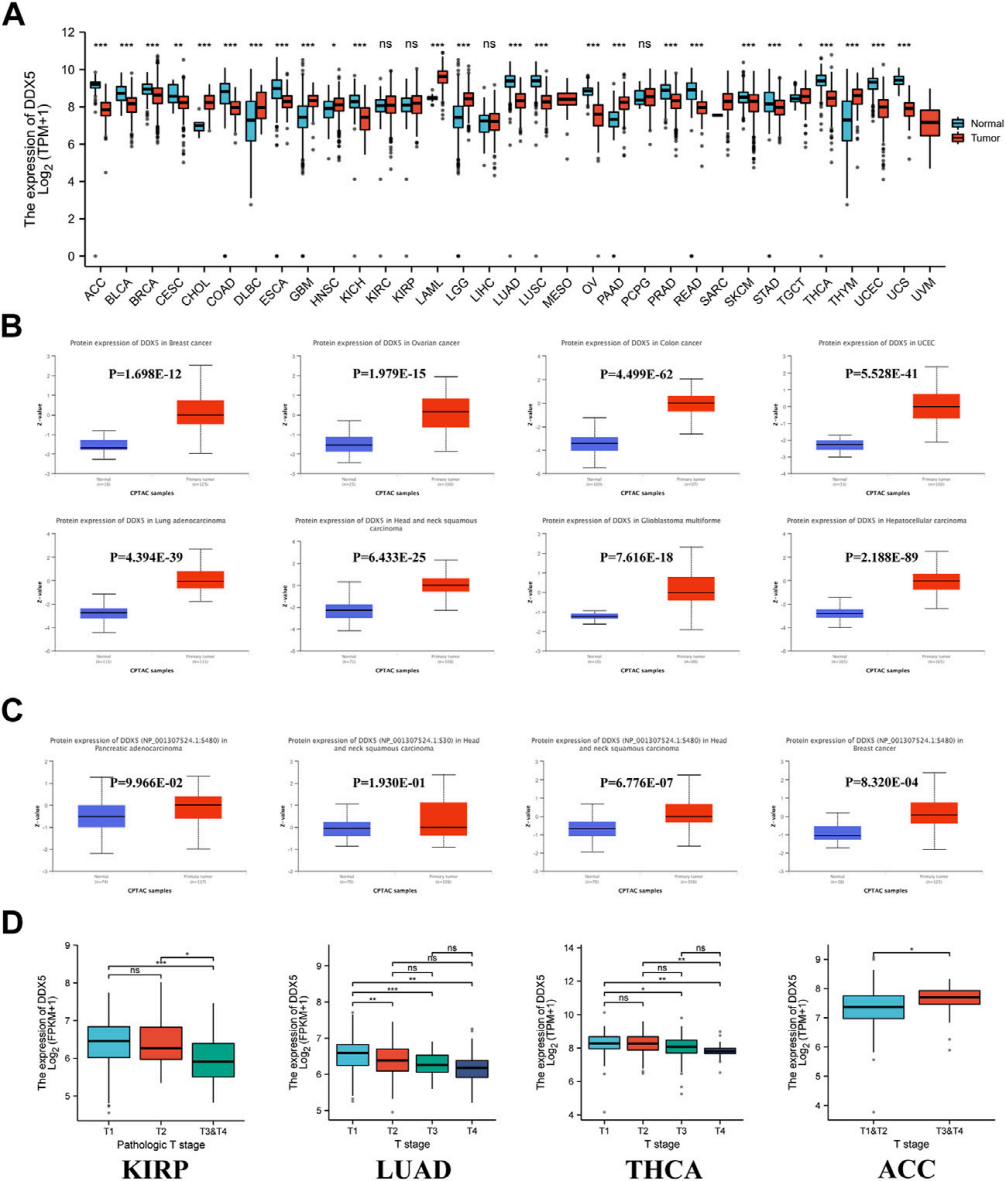
DDX5 gene expression levels in various cancers and pathological phases. **(A)** The protein expression of the DDX5 gene in different cancers, analyzed by UALCAN. **(B)** The protein phosphorylation level of the DDX5 gene in different cancers, analyzed by UALCAN. **(C)**The expression levels of the DDX5 gene analyzed by pathological T stages (T1, T2, T3, and T4) in the type of KIRP, LUAD, THCA, ACC in the TCGA project. The box plot data were provided. ns, no statistical significance, *p < 0.05; **p < 0.01; ***p < 0.001 **(D)**.

The overall scheme of our study is depicted in Figure 1. The expression of DDX5 mRNA in 33 cancer types was compared to that of matched normal tissues based on the data retrieved from TCGA. The results demonstrated that DDX5 mRNA was differentially expressed between tumor and matched normal tissues in 27 different cancer types, with the exception of the malignancies for which data pertaining to matched normal tissues were absent. The expression of DDX5 mRNA was particularly high in eight different types of cancer, including lymphoid neoplasm diffuse large B cell lymphoma (DLBC), glioblastoma multiforme (GBM), acute myeloid leukemia (LAML), brain lower grade glioma (LGG), pancreatic adenocarcinoma (PAAD), thymoma (THYM) (Figure 2A, *p* < 0.001), head and neck squamous cell carcinoma (HNSC), and testicular germ cell tumors (TGCT) (Figure 2A, *p* < 0.05). In contrast, the expression of DDX5 mRNA was downregulated in 17 types of tumors, including adrenocortical carcinoma (ACC), bladder urothelial carcinoma (BLCA), breast invasive carcinoma (BRCA), colon adenocarcinoma (COAD), esophageal carcinoma (ESCA), kidney chromophobe (KICH), lung adenocarcinoma (LUAD), lung squamous cell carcinoma (LUSC), ovarian serous cystadenocarcinoma (OV), prostate adenocarcinoma (PRAD), rectum adenocarcinoma (READ), skin cutaneous melanoma (SKCM), stomach adenocarcinoma (STAD), thyroid carcinoma (THCA), uterine corpus endometrial carcinoma (UCEC), uterine carcinosarcoma (UCS) (Figure 2A, *p* < 0.001), cervical squamous cell carcinoma, and endocervical adenocarcinoma (CESC), compared to that of matched normal tissues (Figure 2A, *p* < 0.01). There were no significant differences in DDX5 mRNA expression among kidney renal clear cell carcinoma (KIRC), kidney renal papillary cell carcinoma (KIRP), liver hepatocellular carcinoma (LIHC), pheochromocytoma and paraganglioma (PCPG), and non-tumor tissues. Owing to the relative scarcity of samples of matched normal tissues of mesothelioma (MESO), sarcoma (SARC), and uveal melanoma (UVM), no significant differences in DDX5 mRNA expression were observed between the tumor and matched normal tissues, which was probably attributed to the limited sample size.

The protein expression data were additionally analyzed using Ualcan’s online CPTAC program. The results of CPTAC revealed that the total expression of DDX5 protein was considerably higher in eight cancer types, including ovarian cancer, breast cancer, GBM, HNSC, UCEC, LUAD, colon cancer, and hepatocellular carcinoma, compared to that of matched normal tissues (Figure 2B, *p* < 0.001). We observed that the gene expression data correlated to protein expression in HNSC, CHOL (Cholangiocarcinoma), and GBM. In contrast, comparison of the gene and protein expression data of the five other cancer types revealed an opposite pattern.

Clinical correlation analysis was additionally performed for determining the association between the expression of DDX5 mRNA and the T-stage of tumors. The different tumor types in TCGA were included in the clinical correlation analysis, and the findings revealed that the expression of DDX5 mRNA differed significantly among ACC, THCA, LUAD, KIRP, and the normal matched tissues (Figure 2D, *p* < 0.05).

Immunohistochemistry (IHC) analysis was performed for corroborating the expression of DDX5 mRNA with the protein expression data at the cellular level, based on data from the HPA database. The data obtained using the HPA020043 antibody revealed that the majority of cancer cells exhibited moderate to high nuclear immunoreactivity. Weak or negative HPA020043 staining was observed in hepatocellular carcinoma and the majority of prostate, lung, and renal cancers. However, the majority of cancer tissues exhibited moderate to strong nuclear staining for the CAB005868 antibody, and a few tissues exhibited additional cytoplasmic staining. The reports in the HPA were congruent with the protein expression data obtained with UALCAN (Supplementary Figure S1).

### Protein phosphorylation analyses

The differences in the phosphorylation of DDX5 protein between primary tumor tissues and normal matched tissues were determined in this study. To this end, the phosphorylation of the DDX5 protein was analyzed in 12 types of cancers using data retrieved from the CPTAC database. The phosphorylation sites in the DDX5 protein were reviewed, and the differences between tumor and normal matched tissues were determined. The phosphorylation of DDX5 increased at the S480 residue in HNSC and breast cancer tissues compared to that of normal matched tissues (Figure 2C, *p* < 0.05).

### Analysis of *DEAD-box helicase 5* gene promoter methylation

**FIGURE 3:**
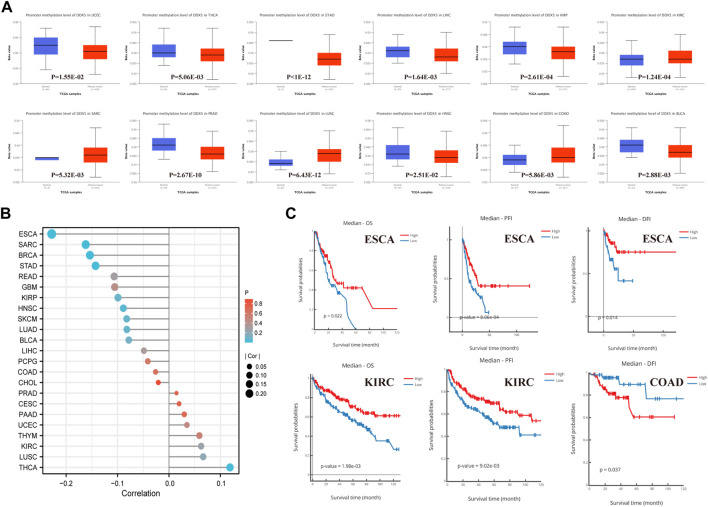
Promoter methylation analysis of DDX5 analyzed by UALCAN, DNMVID, and R. Promoter methylation level of DDX5 across 12 types of tumors in TCGA project **(A)**. The correlation between promoter methylation level and carcinogenesis of different tumors **(B)**. Correlation of DDX5 promoter methylation level with cancer survival prognosis **(C)**.

Analysis of the methylation levels of the *DDX5* gene promoter using the UALCAN dataset revealed the potential function of *DDX5* across all cancer types. The methylation levels of the *DDX5* promoter were significantly lower in eight types of cancer, including STAD, KIRP, PRAD (Figure 3A, *p* < 0.001), THCA, LIHC, BLCA (*p* < 0.01), UCEC, and HNSC compared to those of matched normal tissues (*p* < 0.05). In contrast, the methylation levels of the *DDX5* promoter were significantly elevated in KIRC, LUSC (*p* < 0.001), COAD, and SARC (*p* < 0.01) compared to those of matched normal tissues.

The results of correlation analysis between the pan-cancer methylation and expression of *DDX5* have been depicted with a lollipop graph (Figure 3B, *p* < 0.001). Of the 23 types of tumors, the expression of *DDX5* was negatively correlated to the promoter methylation levels in five tumor types, including BRCA (*p* = 5.47E-06, r = −0.153693), ESCA (*p* = 2.70E-03, r = −0.228029), SARC (*p* = 8.53E-03, r = −0.161896), STAD (*p* = 8.72E-03, r = −0.142683), and HNSC (*p* = 4.27E-03, r = −0.088734); however, the expression of *DDX5* and the promoter methylation levels were positively correlated in THCA (*p* = 2.70E-03, r = 0.118651).

Kaplan-Meier survival analysis, including analysis of the OS, PFI, and DFI, was performed for determining the correlation between the methylation levels of the *DDX5* promoter and patient prognosis. In ESCA, a higher level of promoter methylation was associated with an improved prognosis for OS (*p* = 0.022), PFI (*p* = 8.06E-04), and DFI (*p* = 0.014) (Figure 3C). In KIRC, a higher promoter methylation level was associated with an enhanced prognosis for OS (*p* = 1.98E-03) and PFI (*p* = 9.02E-03) (Figure 3C). However, a higher level of promoter methylation indicated a poor prognosis for DFI in COAD (*p* = 0.003) (Figure 3C).

### Survival analysis

**FIGURE 4:**
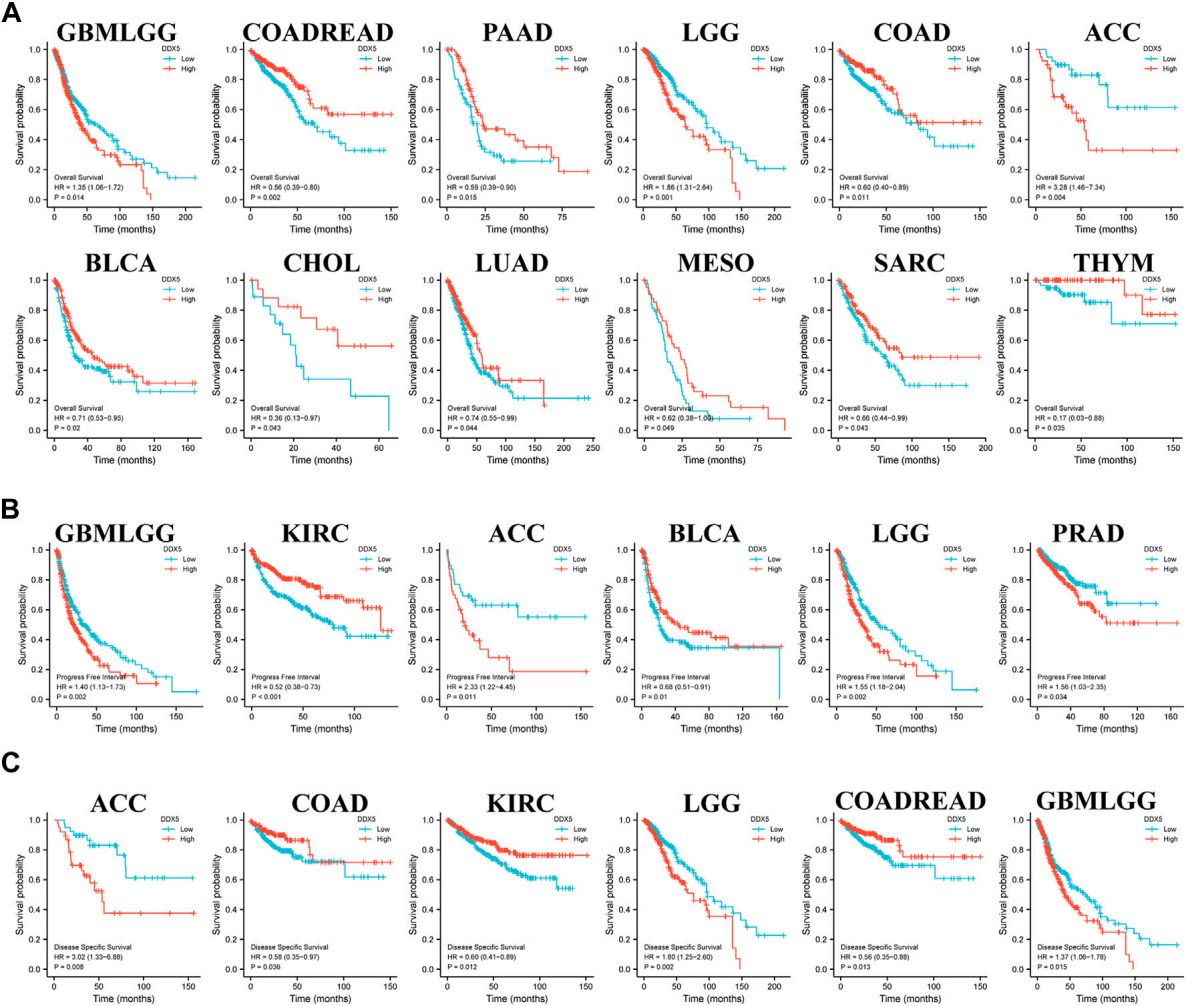
Correlation of DDX5 gene expression with cancer survival prognosis in the TCGA. R was used to analyze the overall survival **(A)**, the progress-free interval **(B)** and the disease-specific survival **(C)** of DDX5 gene expression in different tumors in TCGA. Significant differences in the results were given for survival plots and Kaplan-Meier curves.

Kaplan-Meier analysis was subsequently performed for determining the association between the mRNA expression of DDX5 and the prognosis of patients across the various cancer types in TCGA. Data pertaining to patient prognosis were retrieved from previous studies (Liu et al., 2018). The cancer tissues were categorized into two groups based on the expression of DDX5 mRNA, namely, the high and low expression groups, based on TCGA data. The results of Kaplan-Meier analysis suggested a correlation between the high expression of *DDX5* and the negative prognosis of OS in GBMLGG (*p* = 0.014), LGG (*p* = 0.001), and ACC (*p* = 0.004) (Figure 4A). The Kaplan-Meier curve suggested a positive correlation between the high expression of DDX5 mRNA and poor patient prognosis in terms of PFI in GBMLGG (*p* = 0.019), ACC (*p* = 0.011), LGG (*p* = 0.002), and PRAD (*p* = 0.015) (Figure 4B). Analysis of the DSS indicated that the higher expression of DDX5 mRNA was correlated with worse poor patient prognosis in ACC (*p* = 0.019), LGG (*p* = 0.002), and GBMLGG (*p* = 0.015) (Figure 4C).

Moreover, poor OS was associated with the low expression of DDX5 mRNA in COADREAD (*p* = 0.002), PAAD (*p* = 0.015), COAD (*p* = 0.011), BLCA (*p* = 0.02), CHOL (*p* = 0.043), LUAD (*p* = 0.044), MESO (*p* = 0.049), SARC (*p* = 0.043), and THYM (*p* = 0.035) (Figure 4A). Poor PFI was associated with the low expression of DDX5 mRNA in KIRC (*p* < 0.001) and BLCA (*p* = 0.01) (Figure 4B), and poor prognosis of DSS in COAD (*p* = 0.036), KIRC (*p* = 0.012), and COADREAD (*p* = 0.013) (Figure 4C).

### Association between immune infiltration and *DEAD-box helicase 5* expression

**FIGURE 5:**
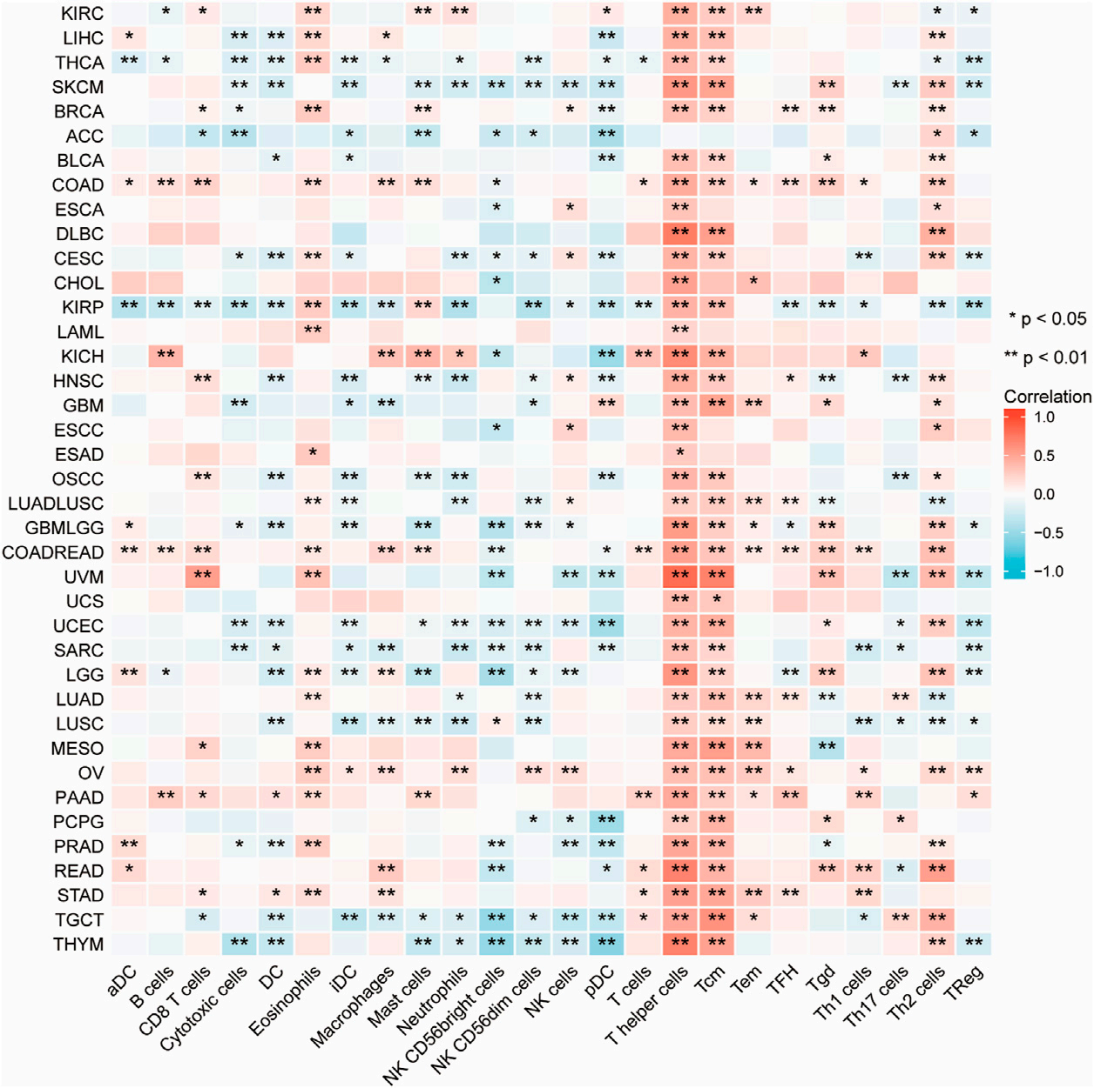
A heatmap about the correlation between immune infiltration of immune cells and DDX5 expression in different cancer. *p < 0.05; **p < 0.01.

**FIGURE 6:**
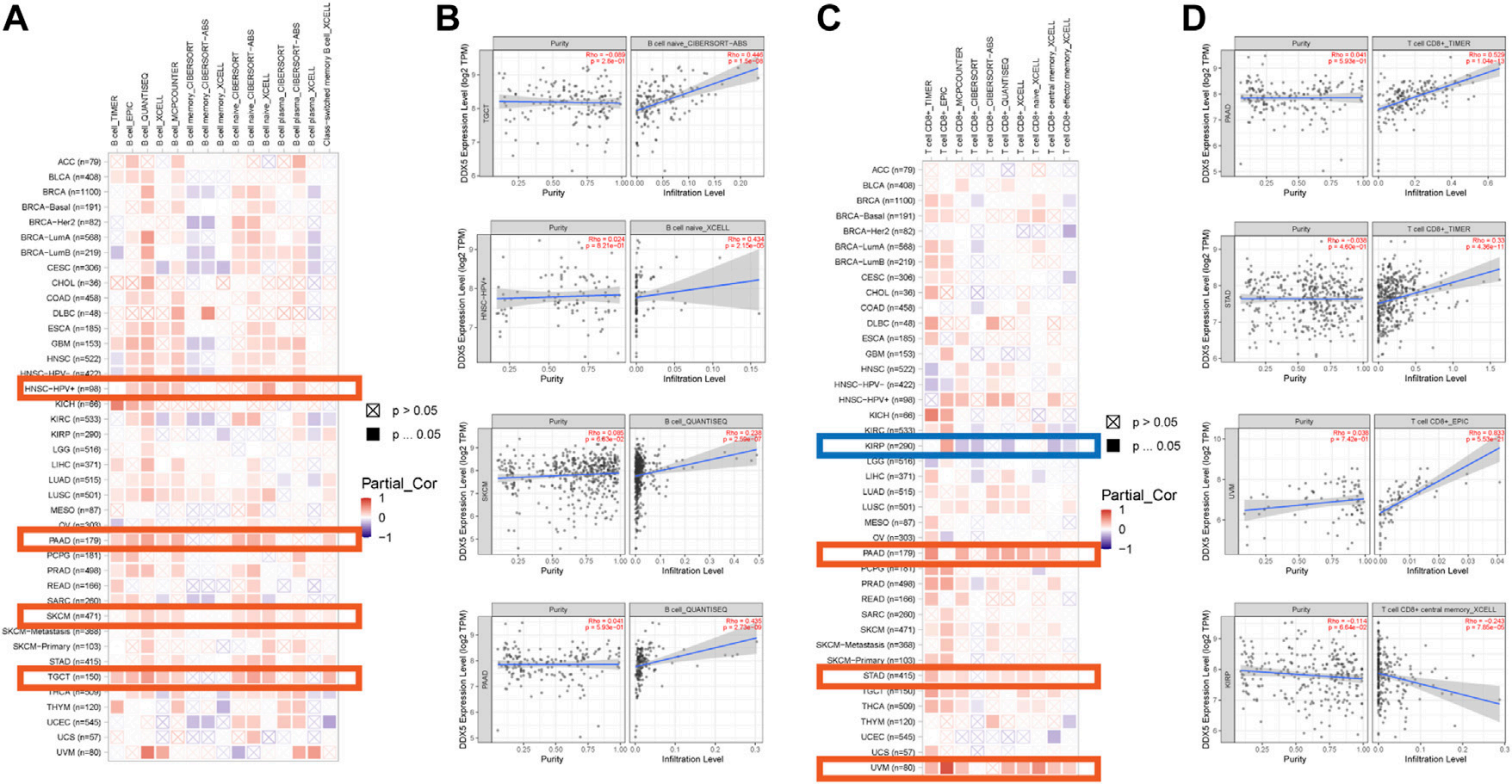
Based on the analysis of 22 immune cell types, the correlation between DDX5 expression and immune infiltration of B cells and CD8^+^ T cells was analyzed. Probe the potential association between DDX5 gene expression levels and levels of B cells **(A)** and CD8^+^ T cells **(C)** infiltration in all types of cancer in the TCGA using various algorithms. B cells’ **(B)** and CD8^+^ T cells’ **(D)** immune infiltration in specific types of tumors.

**FIGURE 7:**
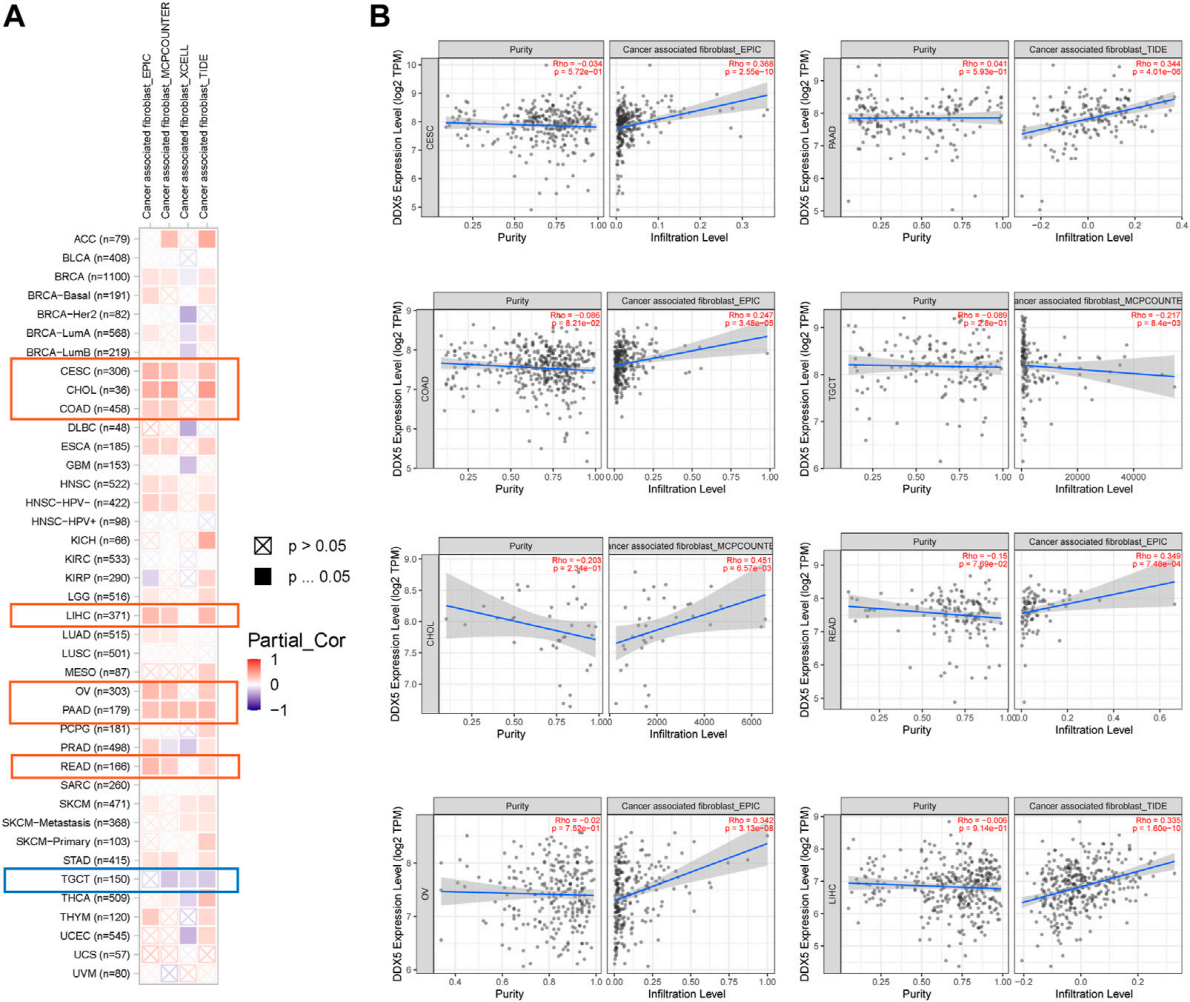
Based on the analysis of 22 immune cell types, the correlation between DDX5 expression and immune infiltration of cancer associated fibroblasts was analyzed. Probe the potential association between DDX5 gene expression levels and levels of cancer associated fibroblasts infiltration in all types of cancer in the TCGA using various algorithms **(A)**. Cancer associated fibroblasts’ immune infiltration in specific types of tumors **(B)**.

Immune cells that infiltrate the tumor are critical to the tumor microenvironment (TME) and are therefore directly linked to tumor initiation, progression, and dissemination. As DDX5 is a promising candidate for triggering the immune response, we used the TIMER 2.0 tool for determining the association between the expression of DDX5 and the infiltration of immune cells. The results demonstrated that the mRNA expression of DDX5 was associated with the infiltration of numerous immune cells, including T-helper (Th), Tcm, Th2, and B cells in 38, 32, 29, and 8 cancer types, respectively (Figure 5). The infiltration of CD8^+^ T cells, cancer-associated fibroblasts, and B cells was positively correlated with the expression of DDX5 mRNA. The results obtained with almost all the algorithms revealed that the immunological infiltration of B cells was significantly correlated with the expression of DDX5 mRNA in HNSC-HPV+, PAAD, SKCM, and TGCT tumors (Figures 6A,B). Additionally, data obtained from TCGA revealed that the expression of DDX5 mRNA was positively correlated with the predicted infiltration levels of CD8^+^ T cells in PAAD, STAD, and UVM tumors, but negatively correlated with CD8^+^ T cell infiltration in KIRP (Figures 6C,D). The infiltration of cancer-associated fibroblasts was positively associated with the expression of DDX5 mRNA in CESC, CHOL, COAD, LIHC, OV, PAAD, and READ tumors but negatively correlated with TGCT tumors (Figures 7A,B).

### *DEAD-box helicase 5* gene alteration data

**FIGURE 8:**
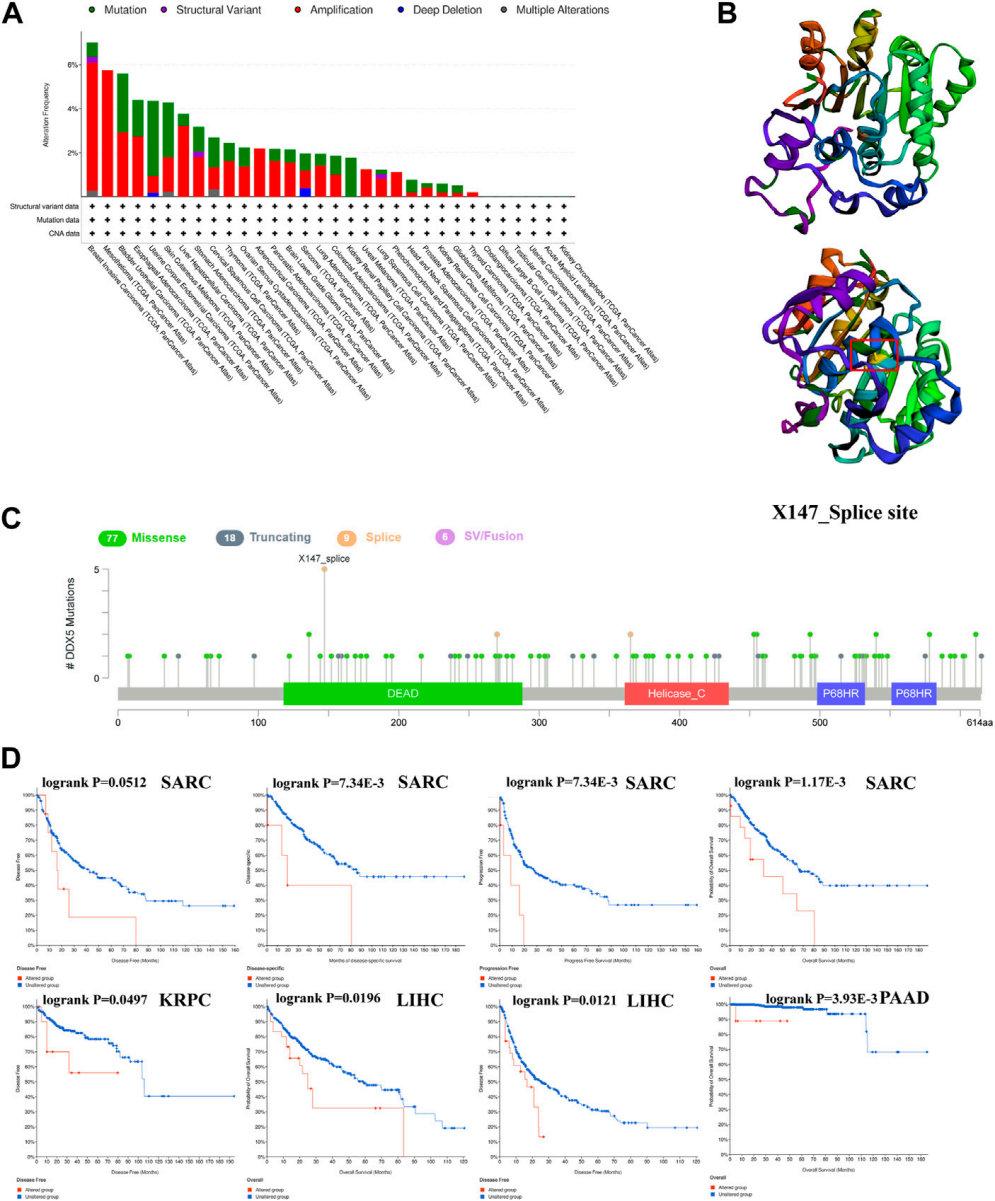
The cBioPortal tool was used to investigate the mutation characteristics of DDX5 in the TCGA database’s various cancers **(A)**. DDX5’s 3D structure and mutation sites **(B)**. The frequency of change with mutation type **(C)**. A cancer survival prognosis on gene alteration level in TCGA tumors **(D)**.

The alterations in the *DDX5* gene in the different tumor types in TCGA were predicted using the cBioportal webtool. Of the 32 types of tumors in TCGA, 26 cancer types showed various degrees of alterations in the *DDX5* gene. It was estimated that the frequency of alteration of *DDX5* was highest in BRCA (>6%), and amplification was the primary form of alteration (Figure 8A). CNAs were the major type of alteration in UCEC, and the frequency of CNAs was approximately 3% (Figure 8A). The frequency of genetic alterations was low in six cancer types. We retrieved the tertiary structure of the DDX5 protein *via* the cBioportal webtool. The X147_splice mutation site was identified in the tertiary structure of DDX5 (Figure 8B). Detailed information regarding the types of genetic alterations of *DDX5*, sites, and number of mutation sites are depicted in Figures 8B,C. Genetic analysis revealed that the *DDX5* gene comprises four domains and a total of 110 mutation sites, of which the most common genetic alteration was a missense mutation. The most frequent X147_splice mutation located in the DEAD domain was detected in five tumors, including LGG, GBM, OV, PRAD, and SARC (Figure 8B), which resulted in a splice site mutation in *DDX5*. The Kaplan-Meier survival analysis was performed for estimating the correlation between survival prognosis and the genetic alteration of *DDX5* in diverse tumor types. The results are depicted in Figure 8D, and the findings indicated that alterations in *DDX5* were significantly negatively correlated with patient prognosis in terms of OS (*p* = 1.17e-3), DSS (*p* = 7.34e-3), and PFS (*p* = 3.33e-3), but not DFS (*p* = 5.12e-2) in SARC. Additionally, the prognosis worsened in terms of OS (*p* = 1.96e-2) and DFS (*p* = 1.21e-2) following alterations in *DDX5* in LIHC. In KRPC, the DFS worsened with alterations in *DDX5* (*p* = 4.97e-2), while the OS worsened in PAAD following alterations in *DDX5* (*p* = 3.98e-3).

### Enrichment analysis of *DEAD-box helicase 5*

**FIGURE 9:**
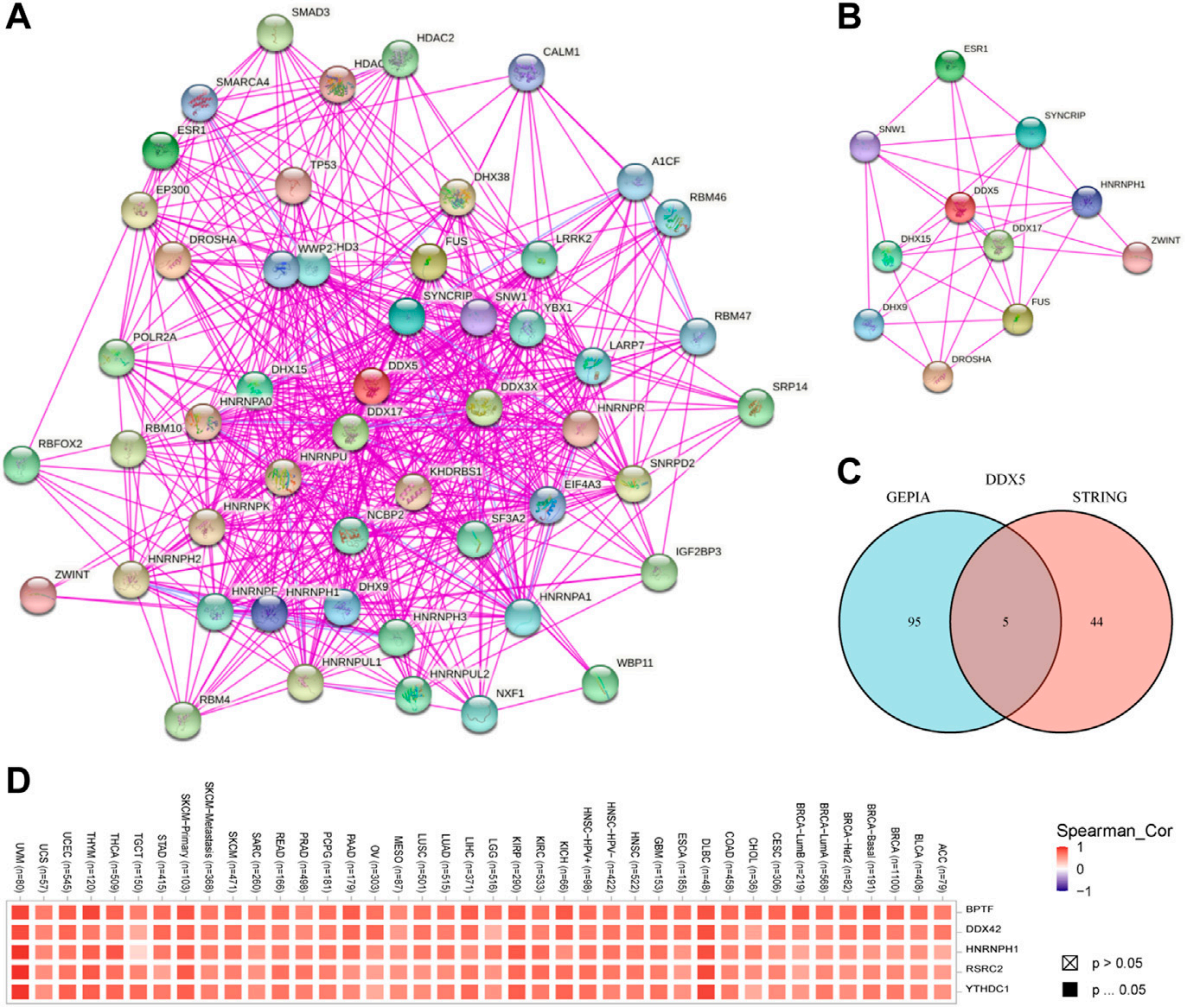
The available experimentally determined DDX5 binding proteins obtained using the STRING tool **(A)** and the main connection **(B)**. A Venn graph between DDX5 binding proteins and DDX5 related genes **(C)**. Expression level of top five DDX5 related genes across TCGA tumors **(D)**.

In order to investigate the molecular processes underlying the effects of *DDX5* in tumor growth, we performed pathway enrichment analyses and analyzed the genes related to *DDX5* expression and the genes that encode DDX5-binding proteins. To this end, we used the STRING webtool for identifying 50 DDX5-binding proteins that had been experimentally verified. The results are depicted in Figure 9A. In order to simplify the network, the 10 most correlated DDX5-binding proteins were selected, as depicted in Figure 9B. The correlation between *DDX5* and the aforementioned five genes, such as *BPTF*, *DDX42*, *HNRNPH1*, *RSRC2*, and *YTHDC1*, was investigated by visualizing the results with a heatmap and identifying the genes that exhibited a positive correlation (Figure 9D). The genes at the intersection of the two aforementioned gene sets were analyzed, and the results demonstrated that the two datasets had five genes in common, namely, *DDX17*, *HNRNPH1*, *HNRNPU*, *HNRNPK*, and *DDX3X* (Figure 9C).

**FIGURE 10:**
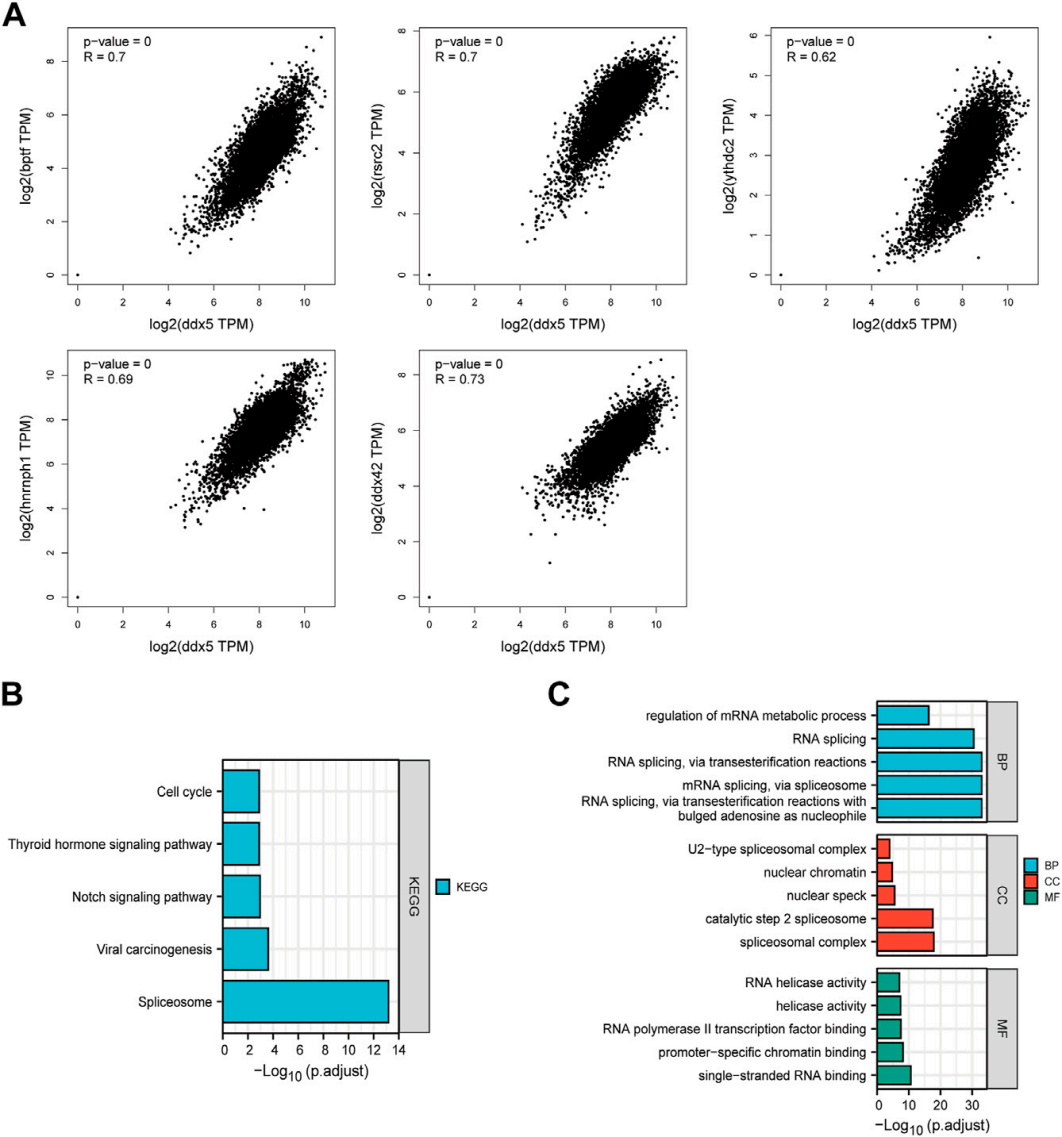
Co-expression of DDX5 and DDX5 related gene. **(A)** KEGG pathway analysis based on DDX5 binding and interacting genes. **(B)** Enrichment analysis of DDX5 related genes cnetplot of molecular function data in GO analysis. **(C)** A cross-analysis of DDX5 binding genes and related genes was performed.

The GEPIA 2.0 webtool was used for identifying the genes that exhibited a positive correlation with the expression of *DDX5* based on TCGA data, and the top 100 positively correlated genes were identified. The expression of *DDX5* was positively correlated with that of *DDX42* (R = 0.73), *BPTF* (R = 0.7), *RSRC2* (R = 0.7), *HNRNPH1* (R = 0.69), and *YTHDC2* (R = 0.62) (*p* < 0.001) (Figure 10A). We finally performed KEGG and GO enrichment analyses of the two aforementioned gene datasets. The results of KEGG analysis revealed that *DDX5* was enriched in the “Spliceosome,” “Notch signaling pathway,” “Thyroid hormone signaling pathway,” “Viral carcinogenesis,” and “Cell cycle” terms (Figure 10B). The results of GO enrichment analysis demonstrated that most of these genes were associated with the pathways or cellular biology of DNA transcription and RNA metabolism. GO enrichment analyses revealed that the genes were enriched in the following biological process (BP) terms: regulation of mRNA metabolic process, RNA splicing, RNA splicing *via* transesterification reactions, spliceosome, and transesterification reactions with bulged adenosine as nucleophile. Additionally, GO analysis revealed that *DDX5* was enriched in the following cellular component (CC) terms: U2-type spliceosomal complex, nuclear chromatin, catalytic step 2 spliceosome, nuclear speck, and spliceosomal complex. GO analysis also revealed that *DDX5* was enriched in the molecular function (MF) terms: RNA helicase activity, helicase activity, RNA polymerase II transcription factor binding, promoter-specific chromatin binding, and single-stranded RNA binding (Figure 10C).

## Discussion

Cancer poses a significant threat to human health and is a primary cause of morbidity and mortality worldwide (Siegel et al., 2022). The development of technologies for the diagnosis and treatment of cancer is the primary focus of current research. With the advent of anticancer therapeutics, including targeted cancer therapy (Huang and Zhou, 2021), the focus of cancer research and treatment has shifted to the molecular and genetic levels (Thomas Boyle and Costello, 1998; Sun et al., 2019).

Previous studies have demonstrated that the *DDX5* protein is a multifunctional DEAD-box RNA helicase and a transcription cofactor that participates in a variety of cellular activities across species (Nyamao et al., 2019). Previous studies have additionally demonstrated that DDX5 is involved in a variety of clinical diseases (Ariumi, 2022; Zhang et al., 2022; Zhao et al., 2022), especially carcinogenesis and the development of cancer (Zheng et al., 2021; Le et al., 2022). The common molecular pathways involved in the function of *DDX5* in carcinogenesis remain to be thoroughly investigated. To the best of our knowledge, there are no published reports on the pan-cancer analysis of *DDX5* to date. We therefore assessed the expression of *DDX5* in 33 cancer types using data from TCGA, CPTAC, and GEO databases, with various webtools and bioinformatics software. The molecular characteristics of *DDX5* expression, genetic alterations, promoter DNA methylation levels, and protein phosphorylation were additionally investigated.

Previous studies have reported that the downregulation of *DDX5* inhibits tumor proliferation (Yang et al., 2006; Shin et al., 2007; Yang et al., 2007; Ponomartsev and Enukashvily, 2015). We observed that of the 33 tumor types in TCGA, the expression of *DDX5* mRNA was significantly reduced in 17 cancer types but significantly increased in eight tumor types. The findings revealed that the expression of *DDX5* mRNA increased in only a small fraction (8) of the 33 cancer types in TCGA. Analysis of mRNA levels is not highly reliable owing to the post-transcriptional regulatory mechanisms. Therefore, the expression of *DDX5* protein was determined in nine cancer types using the CPTAC dataset. The results indicated that the levels of *DDX5* protein were significantly elevated in eight different cancer types. Of these, the mRNA expression of *DDX5* was increased in only three cancer types, indicating the occurrence and significant role of post-transcriptional regulation in the expression of *DDX5*. The mRNA and protein expression of *DDX5* was consistent in GBM, CHOL, and HNSC, and the high expression of *DDX5* was associated with the occurrence of cancer.

Analysis of the relationship between the expression levels of *DDX5* mRNA and tumor progression revealed that the prognosis worsened following the increased expression of *DDX5* mRNA in various cancer types. Kaplan-Meier survival analysis of TCGA data revealed that a higher expression of *DDX5* mRNA was correlated with a negative prognosis in certain cancers, including LGG, GBMLGG, and ACC, and an improved prognosis in other cancer types, including COADREAD and KIRC. The findings suggested that *DDX5* could serve as a viable pan-cancer prognostic biomarker in these tumor types. We additionally identified a correlation between the expression of *DDX5* mRNA and T-stage in certain tumor types. The mRNA expression of *DDX5* decreased in patients with LUAD, BRCA, PRAD, SARC, and THYM with T-stage progression. These findings provide strong evidence that *DDX5* can serve as a biomarker for assessing tumor prognosis.

This study is the first to demonstrate a possible association between the expression of *DDX5* mRNA and the infiltration of immune cells across all cancer types in TCGA. We observed that the infiltration levels of Tcm and Th cells was high in all the tumors at high expression levels of *DDX5*, which suggested that the *DDX5* gene plays a potential role in the tumor immune microenvironment. Th cells, and especially the balance of Th1 and Th2 cells, play a crucial role the immune microenvironment of tumor cells. It has been reported that an increase in the population of Th2 cells increases the risk of tumor invasion and immune escape (Shurin et al., 1999; Lin et al., 2020). The results of this study demonstrated that a high expression of *DDX5* was associated with increased Th2 cell infiltration in several tumors, suggesting an increased risk of immune escape in these tumors. We observed a positive correlation between the infiltration of Tcm cells and the expression of *DDX5*; however, the infiltration of Tcm cells and that of Tem cells were not positively correlated. The findings led us to speculate that the conversion of Tcm cells into Tem cells is possibly inhibited by certain underlying mechanisms. The results demonstrated that the expression of *DDX5* mRNA was associated with the infiltration of B cells, CD8^+^ T cells, and cancer-associated fibroblasts in several cancer types. The presence of B cells is linked with better clinical outcomes in various malignancies (Tsou et al., 2016). Our immune infiltration analysis suggested an increase B cell infiltration for HNSC-HPV+, PAAD, SKCM, and TGCT, partly matched better prognosis in HNSC and PAAD. CD8^+^ T cells have anti-tumor effects. Based on our findings, CD8^+^ T cell infiltration was higher in PAAD, STAD, and UVM and lower in KIRP. Deleting Notch2 from CD8^+^ T cells reduces the efficacy of immune response against tumors, whereas activating the NOTCH pathway may boost immune response. Through gene enrichment analyses, we found evidence that the *DDX5* plays a role in the Notch pathway, thus may upregulate immunological activity of CD8+T cell. The significance of the functions of *DDX5* in the prognosis of cancer and tumor immunity require further investigation in future studies.

The results of enrichment analysis revealed the possible role of *DDX5* protein by integrating information on the *DDX5*-binding proteins and the genes associated with *DDX5* expression across the different cancer types. The results of KEGG analysis revealed that *DDX5* was most significantly enriched in the “Spliceosome,” “Notch signaling route,” “Thyroid hormone signaling pathway,” “Viral carcinogenesis,” and “Cell cycle” pathways, which was consistent with the results of previous studies. The Notch signaling pathway is associated with breast, colorectal, prostate, central nervous system, and lung cancers (Yuan et al., 2014), and is responsible for 60% of acute T lymphoblastic leukemias/lymphomas (Lin et al., 2013). The findings of the present study are consistent with those of a previous report which demonstrated *DDX5* is involved in the co-activation of the oncogenic Notch signaling pathway (Tosello and Ferrando, 2013). There is conclusive evidence that *DDX5* inhibits the DNA replication and biosynthesis in the hepatitis B virus in viral carcinogenesis (Mani et al., 2020; Sun et al., 2022) and interacts with the NS5B protein of the hepatitis C virus (Goh et al., 2004; Dutta et al., 2012). These findings suggest that *DDX5* plays a potential role in the emergence of liver cancer. As a nucleocytoplasmic shuttling protein (Wang et al., 2009), *DDX5* regulates the cell cycle by controlling the expression of p53 to induce DNA damage and cell cycle arrest, which prevents apoptosis and induces cellular survival (Nicol et al., 2013).Analysis of the co-expressed genes of *DDX5* revealed that *DDX5* and its co-expressed genes were significantly positively associated in various cancer types. The aforementioned data strongly suggests that *DDX5* can be exploited as an immunotherapeutic target against cancer.

This study is the first to investigate the association between the methylation levels of the *DDX5* promoter and the incidence of cancer. We observed that the expression of *DDX5* was associated with DNA methylation, and the methylation levels of the *DDX5* promoter may therefore serve as a diagnostic marker of patient prognosis, especially in ESCA. We investigated the degree of *DDX5* protein phosphorylation in 12 cancer types, and the results demonstrated a high degree of phosphorylation at the S430 locus in primary tumors compared to normal matched controls in breast cancer and HNSC, which contributes to the diagnosis and prognosis of cancer.

The feature of *DDX5* is typical in some specific cancers. PAAD is an example. The mRNA expression of *DDX5* upregulated in PAAD. Upregulated mRNA expression of *DDX5* suggests positive correlation with infiltration of Th1, B cells, and CD8^+^ T cells, indicating better prognosis. The OS curves showed a better prognosis with upregulated mRNA expression of *DDX5*. We see a probable link *via* these analyses, suggesting *DDX5* as a potential effective target in PAAD. Results of prognosis analysis are typical GBMLGG and ACC. Three survival indicators, namely, OS, PFI, and DSS, indicated a poor prognosis with upregulated mRNA expression of *DDX5* in these two cancers. We noticed increased infiltration of Th2 in GBMLGG and ACC. The immune escape mediated by Th2 may influence the prognosis. However, further experiments are needed on these points.

In conclusion, this study was the first to perform a comprehensive pan-cancer analysis of *DDX5*, and the findings revealed a positive correlation between the expression of *DDX5* and clinical prognosis, DNA methylation, immune infiltration, tumor mutation, protein phosphorylation, and protein interaction network. The results obtained herein can aid in elucidating the role of *DDX5* in carcinogenesis, and suggest that *DDX5* can serve as a potential biomarker in several cancer types.

## Data Availability

The original contributions presented in the study are included in the article/Supplementary Material, further inquiries can be directed to the corresponding author.

## Supplementary material

SUPPLEMENTARY FIGURE S1 IHC results acquired from HPA database, show different DDX5 protein expression between normal tissues and tumor in different types of cancer. Antibody HPA020043: Breast cancer , ovarian cancer **(C)**, colon cancer **(E)**, LUAD **(G)**, GBM **(I)**, LIHC **(K)**; Antibody CAB005868: Breast cancer **(B)**, ovarian cancer **(D)**, colon cancer **(F)**, LUAD **(H)**, GBM **(J)**, LIHC **(L)**.
