# Supplementary figures and images for: Pan-cancer analysis of the prognostic and immunological roles of DEAD-box helicase 5 (DDX5) in human tumors
Source: Front Genet. 2022 Oct 13;13:1039440. doi: 10.3389/fgene.2022.1039440 (PMC9606813; doi:10.3389/fgene.2022.1039440)

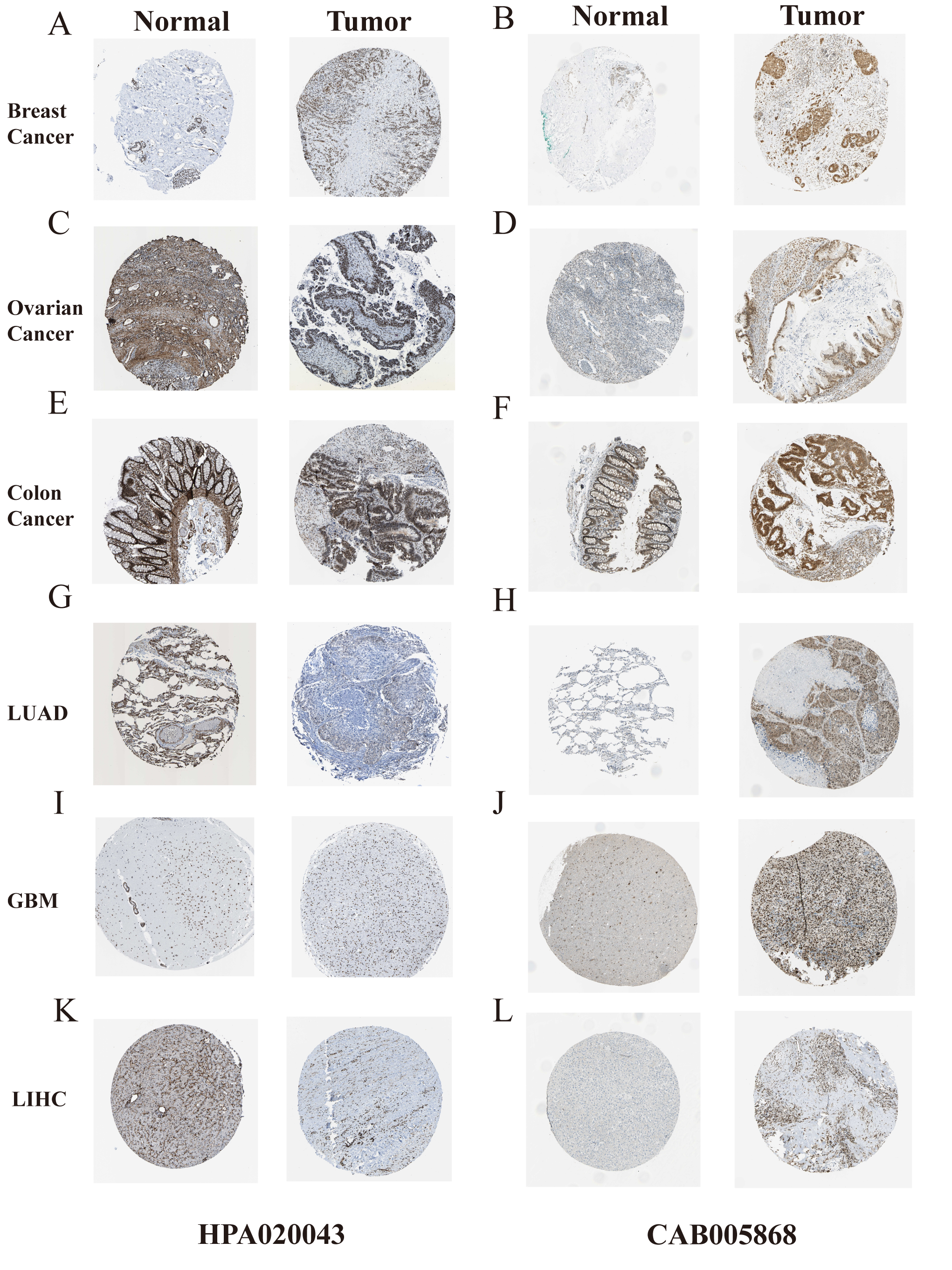

Supplement: Supplementary file 1 [file Image1.TIF]
